# A structured approach to integrating mental health services into primary care: development of the Mental Health Scale Up Nigeria intervention (mhSUN)

**DOI:** 10.1186/s13033-018-0188-0

**Published:** 2018-03-27

**Authors:** Julian Eaton, Oye Gureje, Mary De Silva, Taiwo Lateef Sheikh, Ekpe Esien Ekpe, Mohammed Abdulaziz, Asiya Muhammad, Yusuf Akande, Uchechi Onukogu, Theo Onyuku, Jibril Abdulmalik, Woye Fadahunsi, Emeka Nwefoh, Alex Cohen

**Affiliations:** 1 Centre for Global Mental Health, London School of Hygiene and Tropical Medicine, London, UK; 2 CBM International, Bensheim, Germany; 3 Department of Psychiatry and WHO Collaborating Centre, University of Ibadan, Ibadan, Nigeria; 4 Wellcome Trust, London, UK; 5 Federal Neuropsychiatric Hospital, Kaduna, Nigeria; 6 Federal Neuropsychiatric Hospital, Calabar, Nigeria

**Keywords:** Mental health, Community mental health services, Primary care, Scaling up, Integration, Low-and middle-income countries (LAMIC)

## Abstract

**Background:**

The treatment gap for mental illness in Nigeria, as in other sub-Saharan countries, is estimated to be around 85%. There is need to prioritise mental health care in low and middle income countries by providing a strong body of evidence for effective services, particularly with a view to increasing international and government confidence in investment in scaling up appropriate services. This paper lays out the processes by which a programme to integrate evidence-based mental health care into primary care services in Nigeria was designed, including a research framework to provide evidence from a robust evaluation.

**Methods:**

This paper forms the first step in the overall process evaluation of the mhSUN intervention, where standard research practice indicates that the intervention, and its development, is clearly documented prior to subsequent evaluation. The report covers the period of programme development and evaluation design, and study site and design was chosen to allow generalisability and practical conclusions to be drawn for service development in Nigeria. In order to design an intervention that was informed by evidence and took into account local context and input of stakeholders, a structured process was followed, including: (1) Engagement of relevant stakeholders for information gathering and buy-in; (2) Literature review and gathering of pertinent evidence; (3) Situation analysis at a national and local level; (4) Model development (using Theory of Change); (5) Ongoing consultation, recognising the iterative nature of Theory of Change, and need for ongoing refinement of complex interventions.

**Results:**

The different sections of the structured approach resulted in outputs that built the necessary components (literature review, situation analysis) for informing the Theory of Change. A Theory of Change map is presented, which includes transparent documentation of the assumptions and logic behind the activities to drive the desired change. In addition, it documents the indicators necessary to measure fidelity and draw conclusions as to hypothesised effects of different mechanisms of action in subsequent evaluation.

**Conclusion:**

In addition to the details of ensuring robust evaluation design, there are a number of considerations that are particular to the context that must be taken into account in programme development, including the relationships between ultimate beneficiaries, implementers, host government and institutions, donors, and programme evaluators. Structured methods from existing frameworks can be drawn upon to use and collate relevant information to maximise the local applicability of a generic evidence base. Theory of Change, with its documented assumptions can form the basis of subsequent evaluation and iterative programme refinement, contributing to a more scientifically valid means of developing mental health programmes for scale up.

**Electronic supplementary material:**

The online version of this article (10.1186/s13033-018-0188-0) contains supplementary material, which is available to authorized users.

## Background

Recent epidemiological work has demonstrated that the burden of disease associated with mental and neurological illness is among the highest for all disorders globally [1, 2]. Despite the high level of disability, more than 85% of people with severe mental illness in low and middle income countries do not receive the care they require [3]. In sub-Saharan Africa, less than 1% of the health budget is typically spent on mental health [4]. This small allocation is in countries where an already low proportion of national budget is spent on health, and is often spent inefficiently, with almost all resources used at tertiary hospital level services that are inaccessible to the majority of people [5].

### Evidence for integration of mental health into health services

Integrating mental health into existing health infrastructure improves accessibility, encourages parity between mental and physical health [6], and reduces stigma associated with using services [7]. However, general health systems are typically extremely weak in low income countries. While a balanced approach to care at all levels is necessary [8], there are particular gaps in decentralised, primary level care. Policy in many countries identifies primary services as the site for first line mental health care, but this is often not implemented [4], and infrastructure and personnel struggle to cope with the extra burden that introducing new work brings [9].

The use of less specialised general health staff to deliver defined tasks such as identification, treatment, delivery of psychological therapies or family psychoeducation—termed ‘task sharing’ or ‘task shifting’—has been demonstrated in several low income settings [10]. If this model is to be effective, the quality of service provision must be assured. Several means of achieving this have been proposed and tested, for example stepped care approaches, where patients are treated at the lowest appropriate tier of services using clear guidelines for intervention, and are referred for more specialist care if they meet certain thresholds, for example if they have complications or do not respond to treatment [11, 12]. In addition, the important role of ongoing collaboration between front-line workers and specialists, with supervision and ongoing support after training has been emphasised in many interventions that have used task sharing [13].

A number of trials have established positive results for these interventions in different contexts, and for a range of conditions: in dementia and schizophrenia [14, 15] and epilepsy [16]. For common mental disorders, systematic review has shown moderate to strong effect sizes for clinical benefit and reduction in disability, for low-cost brief psychological interventions delivered by general health workers [17], and stepped care approaches [18].

In recent years, evidence-based resources have become increasingly available to address historic disparities [19] in mental health delivery, for example WHO’s mental health Gap Action Programme (mhGAP) Intervention Guide [20]. There is now good evidence to show effectiveness of increasingly well-defined intervention models, and in a small number of cases, these resources, and the global advocacy for increased investment in mental health are being adopted by national governments, for example in India, China [21] and Ethiopia [22]. However, the scientific underpinning for scale up of services that have been demonstrated at district or regional level remains relatively weak. Several large programmes are now under way to address this, for example through such multi-country programmes as PRIME [23] and Emerald [24].

### Rationale, aims and objectives

Nigeria has been a site for ongoing research in this process, including by collaborators in the research outlined in this article, mainly focusing on system strengthening, and support for self-advocacy by service users [25–27]. Despite the relative volume of research in this field in Nigeria, to date, there has been little commitment to strengthen mental health services from the national (Federal) Government. This reflects well recognised challenges in changing political will in order to effect policy change and mobilise resources [28].

A decision was therefore taken by a number of actors in the country to develop a programme with the aim of contributing towards progressive reform by demonstrating efficacy of integrating mental health into primary care in Nigeria, and to produce results that would be generalizable for sub-Saharan Africa that share many of the structural and resource characteristics of Nigeria. The objectives of the Mental Health Scale Up Nigeria (mhSUN) programme were (1) to develop a model for integration of mental health into primary care in Nigeria that is evidence-based, appropriate to the local context, feasible, accessible, and acceptable to those using the service and providing the service (the focus of this paper); (2) to evaluate the service, focusing particularly on key processes for successful implementation, as well as broad outcomes such as coverage, efficacy and user acceptability, and; (3) to use the results to advocate for service reform and investment by presenting convincing evidence, in an accessible and persuasive format to key decision-makers.

The research associated with the programme aims to evaluate how evidence-based interventions might be utilised appropriately in a particular setting.

## Methods

This paper focuses on the development of an intervention model for the mhSUN programme, which from the outset also sought to establish a suitable framework for evaluation and research. It describes the structured approach that was utilised, which was itself drawn from best practice in similar programmes.

The development of a suitable model for service integration involves a structured process of information gathering and consultation with partners and other stakeholders, in order to align international and local evidence with local needs. We followed a number of key sources for guidance in this process, including the MRC guidance on developing and evaluating complex interventions [29, 30]. In addition, there are a range of resources for project planning in the grey literature. In this case we used the CBM Inclusive Project Cycle Management and Multi-Year Planning tools [31], which have a particular focus on inclusion of people who will be using services. Within the field of Global Mental Health, there are a number of examples of such structured programme development, often as a precursor to trials [32, 33].

Several key issues emerged from this guidance and experience, which we incorporated into our methodology.

*First* It is important to emphasise local expertise and allow this to feed into local adaptation of a consensus (international) evidence base. Stakeholder consultation involved identifying relevant groups, including service users, and facilitating means of collating their perspectives, for example through questionnaires and workshops. Theory of Change was a useful means of documenting their perspectives.

*Second* Sustainability is often inadequately considered in research projects which tend to be shorter-term, and do not have ongoing service provision as their prime purpose. This is a key weakness in generalisability of much trial design. This means that while such models might be *replicated* in a similar context, there are additional factors that if not engineered into the model at an early stage, might render a model with demonstrated efficacy in a trial setting, difficult to *scale up*. This issue is well recognised as a key limit to the current evidence-base, and alongside the problem of funding tending to follow relatively short cycles, is one reason behind the fact that there are relatively few examples of interventions taken to scale. In this case, we adopted an approach which explicitly referenced this issue [34], emphasising engagement with key stakeholders, establishing buy-in at an early stage, and establishing systems of governance that fostered ongoing support.

A third weakness in traditional research trial design that also acts against generalisability and scalability is the degree to which the local environment is amended in order to facilitate fidelity to a model. The intense scrutiny, heavy personal and financial investment, and focus on outcomes, inherent in Randomised Controlled Trials tend to reduce relevance to real-world environments. More naturalistic research methods would be more likely to result in realistic results that can be replicated in less intensely managed and monitored settings. In addition, there is a need for application of implementation science methods alongside the effectiveness trials of which there are now a relatively large number. As a response to these issue, the mhSUN intervention, and its evaluation, was designed to focus on pragmatic, real-world evaluation methods while ensuring scientific rigour. This is in keeping with the objective to provide a model that is not only demonstrably effective, but that can be used practically in the field to meet the growing demand by governments and donors for quality but practical routine monitoring and evaluation, reflecting the available human and financial resources in implementation settings as opposed to research.

Based on these principles, the following stages were followed:

1. *Initial engagement* with partners to gain consensus on aims, scope of the project and desired outcomes. In addition to developing a fundable proposal and establishment of formal partnership structure and contracts, this is an opportunity to gain political buy-in and support [35, 36].
2. *Literature review* In order to understand potential components of an intervention model, and to describe Nigeria’s health and mental health system, several sources were consulted, including:
  Systematic reviews of programme evaluations, and relevant review articles.
  Published evaluations of programmes providing mental health care in low income settings.
  Evidence-based guidelines related to service reform. WHO mhGAP materials were explicitly referenced as the Nigerian government has adopted these as part of national MH Strategy.
  Government and inter-governmental sources related to health services and governance.
  Unpublished programme evaluations, focusing on low income settings, particularly in Nigeria and West Africa, accessed online [6] and through links with implementers in Nigeria.
3. *Situation analysis* to understand the policy context and political environment to guide plans for advocacy towards replication of services, and information about the local population characteristics, resources available, and cultural beliefs. This was carried out using a template developed for the purpose, based on domains derived from the WHO Assessment Instrument for Mental Health Systems (AIMS) [37] (for national level factors like policy and legislation frameworks), the Case Study Methodology [38] field evaluation questionnaires, and PRIME Situation Analysis Tool [39] for a more fine-grained analysis of local health and other sector services.
  The templates were populated at national level using a variety of data sources, including the WHO Mental Health Atlas [4] for information on the mental health system, and online databases such as UNICEF UNDP, and DfID for basic population and demographic data. Nigeria is well served compared to many surrounding countries for national-level data, including epidemiological studies in mental health, and through the National Bureau of Statistics [40]. At State-level and Local Government Area (LGA) level (roughly equivalent to districts in other countries), local researchers completed the templates by accessing local information sources at state level, interviewing relevant experts, and visiting communities to meet with stakeholders. This allowed for documentation of government and civil society and informal services related to the variety of needs that people affected by mental conditions might have, and initial documenting of local beliefs and cultural practices related to mental health in the communities to be served. Such issues would be explored further during training, awareness-raising and other community and stakeholder engagement exercises during the programme.
4. *Model development workshop* with partners, stakeholders and invited experts, to develop a service model and research plan using literature review, situation analysis, and the experience of partners. This was done using Theory of Change (ToC), an increasingly respected method for exploring and documenting the factors that contribute to how and why an intervention achieves the desired impact [41]. ToC is a participatory process of exploring processes for change, which both develops an intervention using the experience and expertise of the participants (while promoting buy-in), and documents key indicators that allow systematic evaluation of processes and outcomes of the intervention. It is particularly suited to generating relevant process evaluation questions, as steps in the service process are clearly documented so assumptions about how one pre-condition leads to an expected outcome can be tested.
5. *Ongoing consultation* with relevant actors was built into the programme model that was developed, for example through State Steering Committees, which included relevant stakeholders in the programme, government health leadership, and service users and carers [42].

### Study setting

**Fig. 1:**
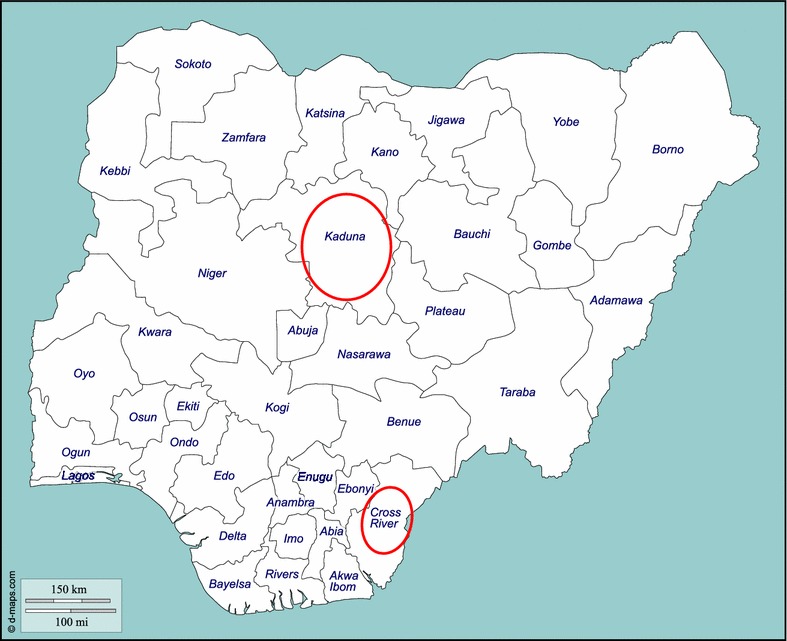
Map of Nigeria, showing the two states of the mhSUN intervention

As an extension of ongoing work between University of Ibadan and CBM International, a scoping exercise for work in integration of mental health into PHC was carried out. As a result, it was decided that the intervention should take place in two sites, one in the South of the country, and one in the North. The inclusion of a site in both settings is useful for generalisability and comparison of contexts, but also has important implications in a country where national unity and broad representation (locally referred to as National Character) are a key factor in any political decision. The States of Cross River and Kaduna were chosen as they represent typical, but distinct cultural, economic, geographical and political realities in Nigeria, allowing exploration of the alignment of the model to these settings, and increased generalisability within and beyond Nigeria. These particular states were also identified for pragmatic reasons as they included well-functioning Federal Psychiatric Hospitals with good expertise and teams who had expressed an interest in developing decentralised services. Each state identified candidate local government areas (3 in Kaduna, 2 in Cross River—based on resources for implementation, and agreement of local authorities), from which health system infrastructure and personnel could be utilised (Fig. 1).

Given the focus on integrating into government structures, local implementation would be overseen by the Federal Neuropsychiatric Hospitals in those two States. These tertiary centres contain sufficient expertise to support local implementation in primary care settings. They were themselves coordinated by the University of Ibadan, allowing a single point of programme management, and technical support for the research component of the programme. PHC services are run through the local (LGA) tier of government in Nigeria, with oversight from State Governments, so it was important that each level was included in local management structures, and involved in planning.

The aim was to effect systems change within government services, so collaboration with government health providers at local and state level was essential. In addition, the Federal Ministry of Health was invited as a partner from the very beginning of the process, in order to foster their engagement (as an ultimate target for advocacy), and gain from their expertise and support.

*The engagement* and consultation process involved visits to key government and health system leaders in the identified states, as well as stakeholder meetings with service users, carers, health workers, NGO partners, and academics. These initial connections were reinforced in the field sites through establishment of local planning groups that subsequently made up the State Steering Committees overseeing programme implementation. An early consequence was that in Kaduna, this led to adoption of a mental health policy by the State Council on Health.

Initial meetings and scoping activities at state level provided a framework within which a funding proposal could be developed by the national partners. The proposal was subsequently funded by the Government of Australia through CBM Australia. See Additional file 1: Appendix S1 for the Organisational Chart of mhSUN Programme.

*The literature review* was carried out (by JE), drawing together relevant evidence for appropriate and effective services in Nigeria. This is summarised in the introduction above, and was presented at the model development/ToC workshop to inform the decisions about the intervention model contents and processes for implementation.

*The situation analysis* was carried out at State level by the local teams. Some information was available in publications or online, but much had to be found through interviews and travel to facilities. Specific information about mental health (prevalence, services, resources), was particularly weak, and either had to be sourced directly, or national data used.

See Additional file 2: Appendix S2 for completed Situation Analysis framework for the two sites, covering local political considerations, demographic situation, health system structure and available resources.

*A model development* and Theory of Change workshop was held in January 2015, to which the main implementing partners, experts in community mental health programme implementation in Nigeria, representatives from the Federal and State Ministries of Health, and international facilitators were invited (a total of 16 people). During the 4 day workshop, the results of the literature review, situation analysis, and interviews with key stakeholders in Nigeria, were presented.

**Fig. 2:**
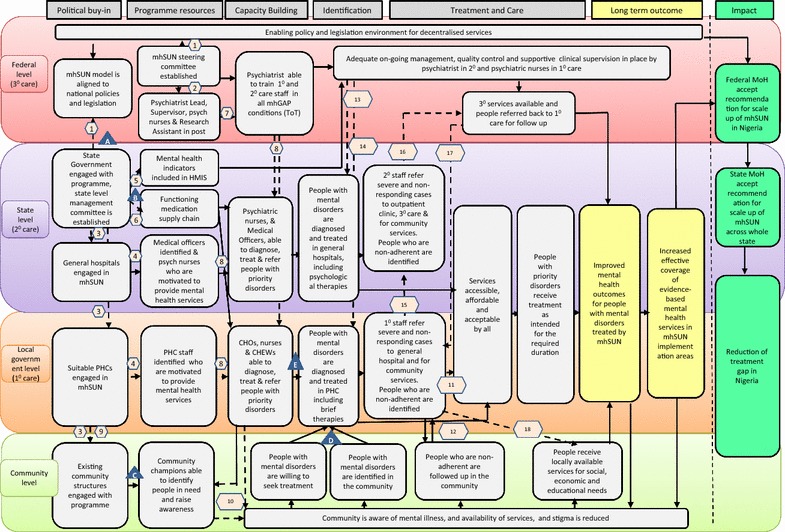
Theory of Change map for mhSUN

The outputs of the model development process included:

1. A Theory of Change map, outlining the logical steps by which certain pre-conditions lead to outcomes and impacts (Figs. 2, 3). This included a more detailed description of each step in tabular form, and indicators to be used in the evaluation to determine whether each step was achieved. See Additional file 3: Appendix S3: Indicators for mhSUN Theory of Change evaluation.

**Fig. 3:**
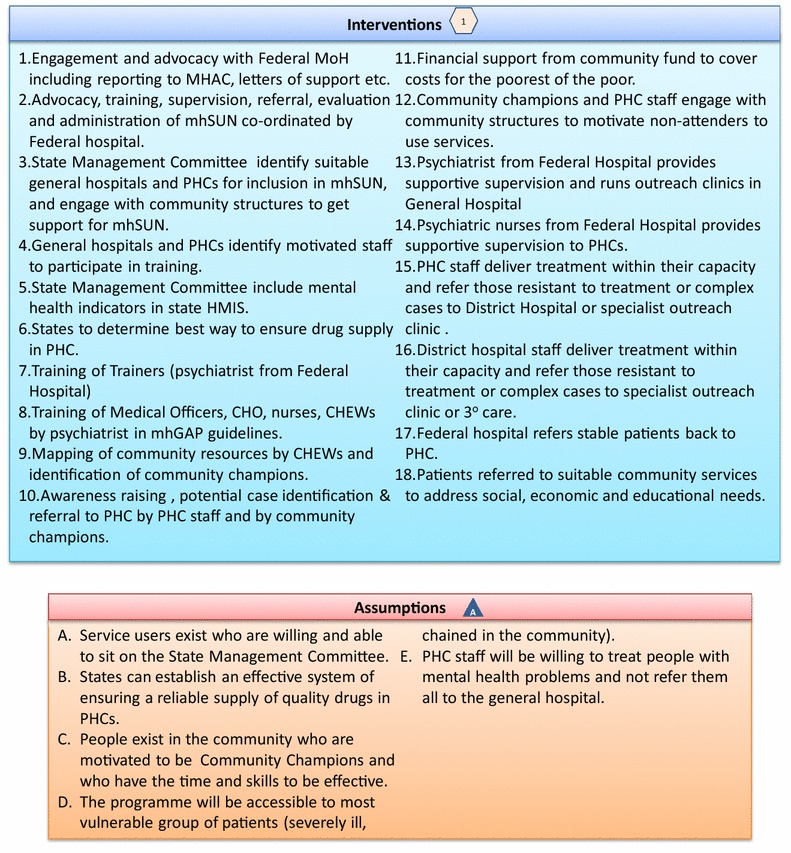
Interventions and assumptions for theory of change
2. A description of the proposed intervention, which was ultimately refined into a Manual of Operations. This is summarised in Box 1: The mhSUN Intervention, and Fig. 4: functions and tasks of different actors.

**Fig. 4:**
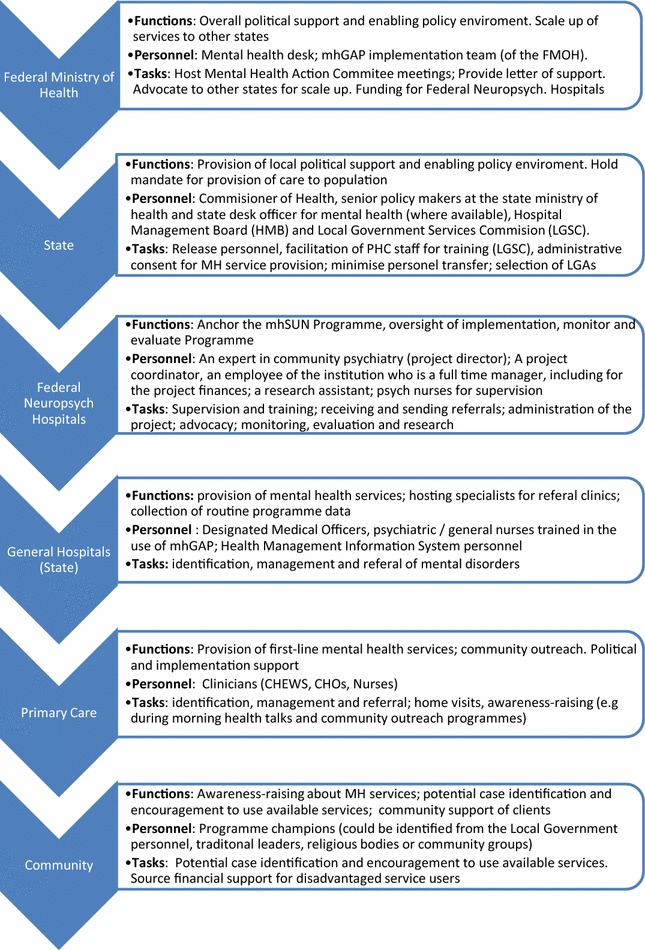
Functions and tasks of different actors

While a common Theory of Change was developed across both sites, it was decided that where circumstances differed, some elements would be amenable to local adaptation. One example of this was ensuring availability of medication. The common problem of lack of effective systems for delivering medication within the state structures was addressed by one site by establishment of a drug revolving fund run by the project, while the other felt that this would not be an acceptable solution, and they would need to work through advocacy with the government to improve availability through the standard supply chains.

*Ongoing consultation and refinement* of the programme is integral to the complex nature of initiating and integrating a new intervention into an existing system. Situation analysis, Theory of Change and intervention implementation are all iterative processes, so these are expected to be revised at key stages in the initial piloting (6 months) and ongoing implementation phases. Specific elements of the Manual of Operations for the service will be reviewed after piloting. For research, data collection, training and competencies of research assistants, data management and secure storage, recruitment issues, and logistics will be considered, and revisions made as appropriate. This period would also serve as an embedding period for the service prior to certain elements of evaluation.

## Results

### Overview of the mhSUN programme model

After following the structured development process as described, a programme model was developed, which was documented through the Theory of Change map, a model description and Manual of Operations. Alongside the implementation of the basic mhSUN intervention (see Box 1), was a deliberate process of engagement with government for advocacy (leading to dissemination of results), including through engagement with local leaders, and support for the National Mental Health Action Committee.

While developing the programme, particularly in the Theory of Change workshop, key areas of debate included:

### Balance between fidelity to an evidence-based model, and resonance with local contexts

This is true on an international level, but in this case, was also an issue in terms of uniformity between sites. As described in the results, the Theory of Change was able to accommodate this. Such points of local divergence might be helpful points for comparison of different intervention components, and demonstrate the flexibility and adaptability of the approach, where ongoing adaptation is recognised as legitimate.

### Engagement with traditional systems

There was consensus that local traditional healers and religious leaders who provided treatment for people who consult them with mental health problems were a key element of pathways to care. Their deep resonance with local explanatory models of mental illness was acknowledged, and a means of engaging with them was included, that would draw upon their experience of effectively addressing concerns of people who used their services, while also addressing concerns that harm is done by some providers, and some interventions used lack efficacy in some cases, resulting in neglect if not identified and alternatives offered.

### Advocacy for resources from government systems

Given the issues of sustainability described above, a clear focus was put in place to not only engage with government early, but to continue effective communication, continuing advocacy at local, State and Federal Government levels, including through provision of accessible evidence from the project. While this remains a focus and commitment, there was a degree of scepticism as to the likelihood of investment in these services, based on past experience, particularly as the economy appeared to be entering challenging times.

### Engaging with communities

It is clear that the health system plays only a small role in recovery and maintenance of mental health, and family and communities have huge impact. While health services have a limited mandate and resources, it was clear that community engagement, through both the existing means used by community mobilisation officers in PHC, and further outreach to communities, was necessary. It was hoped that this might be one means of improving the historically extremely low follow-up rates of patients after initial presentation (usually during crisis).

### Box 1: The mhSUN programme model

*Primary and secondary services will integrate a basic package of mental health care* based on the mhGAP Intervention Guide. This has been previously adapted for the Nigerian context [43], and provides practical, evidence-based guidance for treatment of 8 priority conditions. Services will be provided by primary health care workers who are mainly nurses, community health officers (CHO) and community health extension workers (CHEWs). CHOs and CHEWs are non-physician health workers who have received 2–3 years of post-high school training specifically designed to prepare them for providing essential first-line health care service close to the community. This training will include a component teaching ‘standing orders’ for mental health, however this is very brief, and there is little follow-up or support resulting in a low level of confidence to use this training.

*Capacity building of local health practitioners* will be provided using the mhGAP-IG training package. Initially, master trainers from the University of Ibadan will train local mental health leaders (Training of Trainers). The Federal Neuropsychiatric Hospitals (FNPH) will then be responsible for initial (base) training, and regular refresher training of personnel.

*Ongoing support and skills development of practitioners* included monthly supervision, and support for complex cases through outreach visits (collaborative care) to each clinic at least every month. A system of referral will be put in place (stepped care), including downward referral from specialist care to community follow-up.

*Governance* will be provided through the established health systems structures, with particular attention to mental health aspects through a Steering Committee, made up of government, health service leaders and staff, community leaders, service users, and programme personnel. In addition, the service is designed to comply with national and state legislation, policy and plans.

*Health systems approach* to ensure all relevant components that contribute to successful services will be addressed; negotiating use of appropriate physical infrastructure (access to a suitable private clinic room in each facility), health financing (including consideration of provider and service user costs), health information systems (integrating mental health indicators where they are absent), medication availability, and interaction between the different levels of service (referral and supervision). The need to travel large distances to see a specialist if referred would generally be avoided through monthly consultation in PHC clinics by visiting specialists, and follow-up improved with deliberate efforts to engage with people missing appointments. As far as is possible, established systems will be strengthened and integrated into, rather than duplicated.

*Community engagement* is essential for ensuring social integration, and providing social support. An awareness programme accompanies establishment of the service, including use of local means of sharing information through existing health system means, as well as use of media, and identification of local ‘champions’ for awareness-raising and community support of clients. In addition, community resources will be mapped to promote access to other sectors for social, livelihood and human rights interventions.

## Discussion

This structured approach to using an international evidence base appropriately in a specific local context is central to Global Mental Health in general, and essential in application of global normative standards like mhGAP in diverse countries. We found that this can be done in a systematic way as has been documented in this paper, and demonstrated by some good practices described in published literature.

Since many of these projects have been rooted in research contexts, however, there are several key factors that we emphasised in the development of this project, namely:

*Strong and meaningful participation at all levels* to promote engagement and ensure good fit to the needs of users of services, and those working in them.

*Respecting and integrating with governance structures and other local systems*. While this tends to be a more challenging and longer process, it is likely to result in more sustained change.

*Avoidance of excessive external resource or technical support* that will not be realistically available after a short pilot or trial phase.

*Analysing local context and organising these and other inputs in a structured way* using a Theory of Change methodology, but one that is iterative should the initial experience of implementation demand adjustment.

## Conclusion

Recent years have seen a significant increase in the number of interventions developed and implemented for priority mental disorders in low- and middle-income settings. There remain a number of criticisms related to the consistency of this process, and the degree to which it reflects good practice related to capturing relevant information, organising it in a structured and theoretically sound way, and paying attention to the expertise and experience of local actors. We have developed a comprehensive process for consolidating international and local evidence, adapting this to local needs through consultation with relevant actors, and designing an intervention rooted in a local context. This approach might be useful for those designing other interventions (including as part of evaluation research). The mhSUN programme itself is now proceeding with implementation of the intervention and evaluating it at scale.

There remains a significant degree of art in the science, however, and examination of the processes through which this implementation occurs should enrich our understanding of effective mental health service strengthening.

## Additional files

**Additional file 1: Appendix S1.** Organisational chart of mhSUN programme.

**Additional file 2: Appendix S2.** Situation analysis of political, demographic and services context in Calabar and Kaduna States.

**Additional file 3: Appendix S3.** Indicators for mhSUN Theory of Change evaluation.
