# Supplementary material for: A structured approach to integrating mental health services into primary care: development of the Mental Health Scale Up Nigeria intervention (mhSUN)
Source: Int J Ment Health Syst. 2018 Mar 27;12:11. doi: 10.1186/s13033-018-0188-0 (PMC5870530; doi:10.1186/s13033-018-0188-0)
Supplement: Supplementary file 1 — Additional file 1: Appendix S1. Organisational chart of mhSUN programme. [file 13033_2018_188_MOESM1_ESM.docx]

Local Collaborating Partners

Key:

Fund flows: Green

Reporting flows: Red

Technical Input: Gold

Relevant System Components

Public Private Partnership

Health System Roles

Implementing Levels

**Federal MOH**

(NCD Dep. / MH Focal Point)

Policy Framework

Coordination and Management

**Government / State actors**

Coordination, leadership, infrastructure

Political support and financing

**Specialist Hospital (Fed)**

FNPH or Teaching Hospital

Human Resources

Health

Information System

**Civil Society / NGOs**

Local, national and international

Advocacy, user empowerment

Capacity building

Medication supply

**General Hospital**

**General Hospital**

**State MOH**

(Dir. PHC / MH Focal Point)

State Policy Framework

Service provision

State MH Stakeholder Committee

State MOH

Health sector supervision and referral (collaborative care)

Inter-sectoral collaboration and referral

Community awareness-raising

**Donors /funding agencies**

Financial and other resources

Training

Referral

Supervision

**LGA Health Department**

(HOD Health)

Service provision

Engage local actors, user groups, traditional systems

**Academic**

Technical guidance

Evaluation and research

Master training

**Primary Care Unit**

**Primary Care Unit**

**Primary Care Unit**

**Primary Care Unit**
