# Supplementary material for: A structured approach to integrating mental health services into primary care: development of the Mental Health Scale Up Nigeria intervention (mhSUN)
Source: Int J Ment Health Syst. 2018 Mar 27;12:11. doi: 10.1186/s13033-018-0188-0 (PMC5870530; doi:10.1186/s13033-018-0188-0)
Supplement: Supplementary file 2 — Additional file 2: Appendix S2. Situation analysis of political, demographic and services context in Calabar and Kaduna States. [file 13033_2018_188_MOESM2_ESM.docx]

**Appendix S2: Situational Analysis**

**National Level**

| **Health System Governance** | Tertiary care, and overall health coordination run by Federal Government (Federal Ministry of Health, and various associated relevant agencies, including National Primary Health Care Development Agency, Community Health Practitioners’ Board of Nigeria). Only services are provided by specialist and teaching hospitals around the country.  Secondary care is the responsibility of State Governments (State Ministry of Health under Commissioner for Health). Services provided in general hospitals (and some state teaching hospitals). Also contribute to coordination of PHC across state (under Director of PHC), and employment of a proportion of staff in PHC across state.  Primary care is provided through Local Government Areas, under a Supervisory Councillor for Health. |
| --- | --- |
| **Legislation, Policy and Plans^[[1]](#footnote-1)^** | National Legislation: None enacted (except outdated colonial law), but draft currently under review. Some provision for people with mental illness in criminal law. No legislation specifically protecting rights of people with mental illness. Nigeria has ratified the Convention on the Rights of Persons with Disabilities (CRPD) and signed the Option Protocol.  National Policy: National Policy for Mental Health Service Delivery (2013); Revised National Health Policy (2004)  National Strategy: Strategic Plan for mhGAP Implementation (2013); National Drug Control Master Plan 2015-2019; Nigeria signed the WHO Comprehensive Mental Health Action Plan 2013-2020 |
| **Prevalence of mental disorder^[[2]](#footnote-2)^** | Lifetime rate of at least one DSM–IV disorder: 12.1% (5.8% 12-month rate)  Anxiety disorders: 5.7% lifetime (4.1% 12-month) – mainly specific phobia  Mood disorder: 4.1% (1.3%)  Substance use: 3.9% (0.8%)  Non-affective psychosis: 2.1% (1.1%)^[[3]](#footnote-3)^  Of those who had seriously disabling disorders, only about 8% had received treatment in the preceding 12 months. |

**State Level**

**Socio-demographic characteristics**

| **Population characteristics** | **Cross River State** | **Kaduna State** | **Notes**  ***Relevance for mhSUN*** |
| --- | --- | --- | --- |
| **1. State Population (2014)^[[4]](#footnote-4)^** | 3,454,379  Male %: 50.88  Female % : 49.12 | 7,853,338  Male %: 51  Female % : 49 | *Coverage calculation*  *Provision of gender-relevant services* |
| **2.Geography^[[5]](#footnote-5)^** | Total area: 22,342km^2^  Largest town: Calabar  Number of LGAs: 18 | Total area: 46,053 km^2^  Largest town: Kaduna Town  Number of LGAs: 23 | *Logistics*  *Patient service use patterns* |
| **3.Age range^[[6]](#footnote-6)^** | 0-14yrs: 38.2%  15-64yrs: 58.7%  >65yrs: 3.1% | 0-14yrs: 46.1%  15-64yrs: 51%  >65yrs: 2.9% | National, 2016:  **0-14 years:** 42.79%  **15-54 years:** 54.09%  >65yrs**:** 3.2%  *Service planning* |
| **4.Ethnicity** | % of different groups (three most common)  Efik 35%; Ejagham 40%; Betwarra 5% (% estimates) | % of different groups (three most common)  Hausa-Fulani; Gbagyi; Baju | *Translation of documents, and awareness materials.*  *Ensure there are staff who speak different languages* |
| **5.Languages** | Main 3 languages  Efik; Ejagham; Bekearra  English and Pidgin English widely spoken | Main 3 languages  Hausa-Fulani (very widely spoken estimated 85%);  Gbagy; Baju (around 60 other groups exist in State)  English less widely spoken than in South |  |
| **6.Religion** | Christian: 75%  Traditional: 24%  Muslim: 1%  Role of religion:  Christianity has been an important role in education and health service provision. The first Christian school opened in 1895. The Church also plays an important social role for many families. | Muslim: 56%  Christian: 25%  Traditional: 19%  Role of religion:  There are few Muslim hospitals providing services as with the tradition of Christian mission hospitals.  Many people turn to Muslim or Christian leaders in mosques or churches when they have illness, particularly mental illness | The census of 1963 collected details on religion, but since then this question has been omitted. These are 1963 figures. Some State borders have changed, but total figures reflect proportions of religions practiced.^[[7]](#footnote-7)^  These represent the first stated religion, but many people also follow traditional belief systems |
| **7.Literacy rate^[[8]](#footnote-8)^** | Overall: 76.6%  Male: 82.5%  Female: 70.0% | Overall: 62.9%  Male: 67.8%  Female: 57.9% | *Need to provide information verbally, including consent*  *Use of leaflets etc* |
| **8. Economic status** | % in poverty (earning <USD 1.25 /day): 33.1%^[[9]](#footnote-9)^  Average monthly income: NGN 5,500^[[10]](#footnote-10)^ | % in poverty (<USD 1.25 /day): 56.5%^i^  Average monthly income: NGN 3,200^j^ | *Travel to services may be a barrier to access*  *Challenge of rehabilitation and inclusion in difficult economy* |
| **9. Employment** | Main types of employment:  Civil service; small business; agriculture, Trading  Many rural people rely on informal economy and subsistence farming | Main types of employment:  Civil service; agriculture; trading  Many rural people rely on informal economy and subsistence farming | National unemployment + underemployment rate^[[11]](#footnote-11)^: 35.2% (Q4 2016)  Youth unemployment + underemployment rate: 56% (Q4 2016)  Unemployment higher in North |

**General health services**

| **10. Governance** | State role: Responsible for public health in the state, secondary health services (eg general hospitals), and coordination of primary health care.  Official objective: “To have the best quality and most accessible health care services that addresses public health issues in Nigeria.” (Kaduna State)  “Sustain Health Services Management will deliver the best possible care to people of CRS, nearest to where they live and work.” (Cross River State) | | | |
| --- | --- | --- | --- | --- |
| **11.Payment system^[[12]](#footnote-12)^** | Means of payment:  Out of pocket: 75%  Government: Federal; 6%; State; 7%; Local Gov; 6%  Insurance: 3%  While there is a national Health Insurance Scheme, to date, only State and Federal Government employees are included | | | |
|  | **Calabar** | | **Kaduna** | |
| **Facility** | **Number in State** | **Notes** | **Number in State** | **Notes** |
| **12.Federal Medical Centre / Federal Teaching Hospital** | 1 | Federal Neuropsychiatric Hospital, Calabar. Specialist psychiatric hospital, teaching hospital  University of Calabar Teaching Hospital; Specialist tertiary hospital, teaching hospital. | 3 | Federal Neuropsychiatric Hospital, Kaduna (FNPH); Tertiary specialist psychiatric hospital  Ahmadu Bello University Teaching Hospital (ABUTH). Includes department of psychiatry  Army Reference Hospital, Kaduna |
| **13.District or State General Hospitals** | 15 total | Staff in State Hospitals:  Doctors: 54  Nurses: 962  Other paramedics: 415 | 30 General Hospitals and 2 Dental centres | Staff in State Hospitals:  Doctors:93  Nurses: 1437  Other paramedics: 839 |
| **14.Primary Health Centres (PHC) and Health Posts (HP)** | PHC: 130  HP: 684 | Staff in PHC:  Doctors: 18  Nurses: 89  CHOs: 72  CHEWs: 1165 | Total PHC+HP: 1015 | Staff in PHC^[[13]](#footnote-13)^:  Doctors: 0  Nurses: 197  CHO: 162  CHEWs: 1325 |
| **16.Private hospitals/ clinics** | 183 | Doctors: 100  Nurses: 130^[[14]](#footnote-14)^ | 533 | Not recorded |
| **17.Others** |  | |  | |
| **Out of state medical facilities visited for MH care** | Popular religious healers, eg JP Joshua in Lagos | | Spiritual Homes in Lagos and others cities.  People from tribal groups tend to go to home state for treatment. | |
| **Traditional/ herbal** | Many traditional healers evoking ancestors and traditional beliefs, use herbs, incantations, potions and prayers  There has been a shift away from traditional practices towards religious (Pentecostal Christian) healers | | Many traditional healers will treat mental illness with herbs and prayer | |
| **Religions (Islamic, Christian)** | Religious Prayer Houses , Healing Homes , and Spiritual Homes run by Churches (mainly Pentecostal), for example The Brotherhood of Cross and Star; Eternal Sacred Order of Cherubim and Seraphim (and many others)  Church Leaders, often called Prophets, Evangelists, Pastors, etc. who have a reputation for healing mental illness. Based on possession by spirits or witches  Chaining, (forced) fasting, and prayer are used. People may be kept in churches or related buildings for many months. | | Popular local Islamic cleric in Islamic Healing Home who also sees patients with substance use disorders. There have been various reports of violation of human right in the centre.  Many Ruqya centers (Islamic centres addressing spirits/possession), who treat mental illness. Numbers not known as not required to register and are not regulated by any government agencies. Methods include recitation of quranic verses to the patients to remove evil spirits (djin). Beating of patients is also employed. | |

**Mental health services**

|  | **Cross River State** | **Kaduna State** |
| --- | --- | --- |
| **18. State Governance** | State Ministry of Health  No existing specific authority for MH governance in State | State Ministry of Health  Policy level collaboration with state (eg Training of 40 Nurses at ministry)  No formal policy or specific authority in place for MH governance |
|  | Level of political commitment: Minimal | Level of political commitment: Minimal |
|  | MH integrated into general health system: No | MH integrated into general health system: No |
| **19. State Mental Health Policy and plan** | Policy; None | Policy; None |
|  | Plan; None | Plan; None |
|  | Legislation: None | Legislation: None |
| **20.Investment in Mental Health** | Federal: Large: FNPH and University of Calabar Teaching Hospital  State: Minimal  LGA: None  Private: Few private clinics | Federal: Large: FNPH and Ahmadu Bello University Teaching Hospital  State: Minimal  LGA: None  Private: Personal investment in 5 private clinics |
| **Human Resources** | | |
| **21.Total Mental health professionals** | Psychiatrists: 36  Psych Nurses: 123  Psychologists: 7  Clinical Social Workers: 15  Occupational therapists: 6  Other MH Professionals: 66  Almost all at Federal Neuropsychiatric Hospital | Psychiatrists: 42  Psych Nurses: 115  Psychologists: 14  Clinical Social Workers in MH: 39  Occupational therapists: 4  Other MH Professionals: 47  Almost all at Federal Neuropsychiatric Hospital |
| **Facilities** | | |
| **22. Mental health service types available** | FNPH Calabar:  In-patient beds: 201  Outpatient services  Outreach: Clinic in Prison  Community/PHC: Support for 2 Local Government Areas – mhSUN)  University of Calabar Teaching Hospital:  Inpatients admitted to general wards, and outpatient services | FNPH Kaduna:^[[15]](#footnote-15)^  In-patient beds: 162 (Adult beds: 132, Child & Adolescent beds: 20,  Emergency Ward: 10)  Outpatient services  Laboratory services; EEG; ECT (Modified)  Community/PHC: Support for 3 Local Government Areas – mhSUN  Ahmadu Bello University Teaching Hospital:  Inpatient services (25 beds,) and outpatients services |
| **23. Other Mental Health Services** | 1 Charitable/NGO: Ladies of the mind; awareness-raising and individual support | 5 private clinics |
| **24. Community-based services** | None | 3 outreach clinics run by FNPH |
| **25. Mental health in state health information system** | None included in State system | Not included in State system |
| **26. Psychotropic medication availability in different levels of system** | PHC: None  General Hosp: Anxiolytics (diazepam)  FNPH: Wide range  Federal Essential Drug List adopted by State (but not available)  Cost for 1 month supply of traditional antidepressants:  NGN 900 (USD 3) | PHC: None  General Hosp: Occasional anxiolytics  FNPH: Wide range  Please list:  Cost for 1 month supply of traditional antidepressants:  NGN 500 (USD 1.8) |
| **27. Psychosocial interventions available** | PHC: Nil  General H: Nil  FNH: Full psychological interventions available. Some occupational therapy (pre-discharge rehab). Social work with limited ability to work in community. | PHC: Nil  General H: Nil  FNH: Full psychological interventions available. Some occupational therapy. Social worker have limited ability to work in community due to lack of resources. |
| **28.Mental health promotion activities** | PHC: Health talks, awareness campaign in some PHCs by Ladies of the Mind charity (scaled up under mhSUN)  General Hosp: Nil  FNPH: Health Education, counselling, awareness talk to public groups. | PHC: Nil  General Hosp: Nil  FNPH: World Mental Health day activities, Health talks by nurses at the OPD and community engagement in the form of radio and television shows by FNPH management |
| **39.Patient costs** | For a 1 month admission: NGN 26,000  For outpatient care (per month): NGN 3,000 | For a 1 month admission: NGN 30,000 to 45,000  For outpatient care (per month): NGN 2,500 – 4,500 |
| **30.PHC Staff attitudes towards working in mental health** | Where FNPH have engaged PHC staff, there is a general change of attitude and awareness towards mental health issues. At present most are reluctant to work in mental health | Initially dubious but there is a general change of attitude and awareness towards mental health issues by PHC staff (where addressed). |

**Non-health resources**

| **Information** | **Cross River State** | **Kaduna State** |
| --- | --- | --- |
| **32.Education; proportion of children in school^[[16]](#footnote-16)^** | Net Attendance Ratio:  Primary: M: 71.4%; F: 75.2%  Secondary School: M: 69.5; F 55.5%  National Statistics imply that very few children with mental disabilities attend school | Net Attendance Ratio:  Primary: M: 58.9%; F: 57.3%  Secondary School: M: 46.7%; F: 38.4% |
| **33.Social welfare services (State)** | Available under the auspices of the state Ministry of Social Welfare  First Lady of the State has some budget for welfare | Ministry of Women Affairs and Social Development  Kaduna State Agency for Aids Control  There are social welfare offices attached to hospitals |
| **34. Other welfare services** | Church: Provision of support to the less privileged, old people Homes, Motherless Babies Homes. Many churches run financial savings/support schemes (eg for funerals)  NGO/civil society: Police Officers Wives Association, Nigerian Army Officers Wives Association, Airforce Officers Wives Association, Rotary Club, Lion’s Club Traditional: Exist at many levels; community organisations, Age Grades | Mosque: Islamic charitable institutions. Part of activity of mosque is to support less privileged  NGO/civil society: Centre for Integrated Health programmes. Kaduna State Adult Schools, Maternal Newborn and Child Health (MNCH) services. Many health-related NGOs have economic supports  Traditional: Exist at many levels; community organisations, Age Grades |
| **35. Livelihood support /programmes** | Mothers Against Child Abandonment (MACA)  A State Fit for A Child (ASFAC)  Small Scale Business Entrepreneurship Training/Loan Scheme by State | National Directorate of Employment (NDE): FNPH is collaborating to create training and support programs.  Centre for Integrated Health Programme (CIHP) have monthly programme for disabled |
| **36. Access to Justice/ Human Rights organisations** | Constitutional rights enshrined in law but few effective mechanisms for realisation. None focus on mental health | Non-governmental Civil Rights Organizations, Women for Justice etc handling molestation and homelessness. None focus on mental health  The Ministry of Health was formally with social development, the component of social welfare is now passed on to Women Affairs |
| **37. Service-user organisations** | None  Self-help group associated with outreach work of CBM Partner programme Edawu in Benue State (across border) | None |

1. WHO. MiNDbank online legislation and policy resource [www.mindbank.info](http://www.mindbank.info) Accessed June 2017 [↑](#footnote-ref-1)
2. Gureje O, Lasebikan VO, Kola L, Makanjuola VA. Lifetime and 12-month prevalence of mental disorders in the Nigerian Survey of Mental Health and Well-Being. *The British Journal of Psychiatry* 2006; 188(5): 465 [↑](#footnote-ref-2)
3. Gureje O, Olowosegun O, Adebayo K, Stein DJ. The prevalence and profile of non-affective psychosis in the Nigerian Survey of Mental Health and Wellbeing. *World Psychiatry* 2010; 9(1): 50-5 [↑](#footnote-ref-3)
4. National, State and Local Government Area Population Figures. [<http://www.population.gov.ng/index.php?id=11>] [↑](#footnote-ref-4)
5. National Population Commission. National Population and Housing Census. Abuja: National Population Commission, 2006 [↑](#footnote-ref-5)
6. National Population Commission. National Population and Housing Census. Abuja: National Population Commission, 2006 [↑](#footnote-ref-6)
7. Ostein P. Percentages By Religion of the 1952 and 1963 Populations of Nigeria’s Present 36 States. Oxford: Nigeria Research Network, 2012 [↑](#footnote-ref-7)
8. NPC. National Population and Housing Census. Abuja: National Population Commission, 2006 [↑](#footnote-ref-8)
9. United Nations Poverty Index, 2015 [↑](#footnote-ref-9)
10. State Planning Commission, Cross River State [↑](#footnote-ref-10)
11. National Bureau of Statistics <http://www.nigerianstat.gov.ng/> Accessed Aug 12^th^ 2017 [↑](#footnote-ref-11)
12. WHO. Health Expenditure Indicators. 2015 <http://apps.who.int/nha/database/Select/Indicators/en> Accessed June 2015 [↑](#footnote-ref-12)
13. Kaduna State Health bulletin, 2012 [↑](#footnote-ref-13)
14. Association of General And Private Medical Practitioners of Nigeria (AGPMPN) Cross River Chapter [↑](#footnote-ref-14)
15. Federal Neuropsychiatric Hospital, Kaduna [www.fnphkaduna.org](http://www.fnphkaduna.org) Accessed Feb 2017 [↑](#footnote-ref-15)
16. NPC, ICF. Nigeria Demographic and Health Survey 2013. Abuja, Nigeria, and Rockville, Maryland, USA: NPC and ICF International, 2014 [↑](#footnote-ref-16)
