# Supplementary material for: A structured approach to integrating mental health services into primary care: development of the Mental Health Scale Up Nigeria intervention (mhSUN)
Source: Int J Ment Health Syst. 2018 Mar 27;12:11. doi: 10.1186/s13033-018-0188-0 (PMC5870530; doi:10.1186/s13033-018-0188-0)
Supplement: Supplementary file 3 — Additional file 3: Appendix S3. Indicators for mhSUN Theory of Change evaluation. [file 13033_2018_188_MOESM3_ESM.docx]

**Appendix S3: Indicators for mhSUN Theory of Change evaluation**

| **#** | **Pre-condition** | **Indicator** | **Evaluation method** | **By whom *** |
| --- | --- | --- | --- | --- |
| 1 | Enabling policy and legislation environment for decentralised services | 1.1 Policy is in place that supports reform and scale up of services | Review of documents, Policy mapping  Interview with FMOH MH Desk Officer | Research project |
| 2 | mhSUN model is aligned to national policies and legislation | 2.1 Model elements reflect policy points | Observation  Interview with FMOH MH Desk Officer  Fidelity measure | Research project |
| 3 | mhSUN steering committee established | 3.1 Steering Committee already established [Binary; Yes/No] | Observation, number of meetings | mhSUN M&E |
| 4 | Psychiatrist Lead, Supervisor, psych nurses & Research Assistant in post | 4.1 Already identified [Binary; Yes/No] | Observation | mhSUN M&E |
| 5 | State Government engaged with programme, state level management committee is established | 5.1 Engagement meetings (1 per quarter)[Binary; Yes/No]  5.2 Management Committee Membership finalised [Binary; Yes/No] | Document reviews (minutes of meetings, letters) | mhSUN M&E |
| 6 | Mental health indicators included in HMIS | 6.1 Minimum set of indicators being collected  a) by programme M&E  b) through mainstream HMIS | mhGAP Indicators  Compare with needs of national reporting, donors, M&E, research  Use experience from elsewhere, eg the Benue CCMHP experience Description of the process to arrive at the end point | Routine HMIS  mhSUN M&E  Research project |
| 7 | Functioning medication supply chain | 7.1 Availability of essential medication at point of clinical contact (proportion available on supervisory visits)  7.2 Prescription patterns (optional add on) | mhGAP monthly monitoring tool – a checklist of medications available on the given day of visit  Facility Case Study | mhSUN M&E  Routine HMIS |
| 8 | General hospitals engaged in mhSUN | 8.1 General Hospital fulfilling role stipulated in model (3 per State plus FNPH) | Letters of support/MOUs  Facility Case Study | mhSUN M&E |
| 9 | Medical officers identified & psych nurses who are motivated to provide mental health services | 9.1 Adequate numbers attend training for model needs (5 per Gen Hospital), making allowance for attrition | Training attendance | mhSUN M&E |
| 10 | Suitable PHCs engaged in mhSUN | 10.1 Adequate number for model and expected coverage (15 per State), which fit set criteria, eg   - High pt flow rate - Adequate staff levels to allow one ‘MH personnel’ always on duty - Adequate geographical coverage | Letters of support/MOUs  Mapping of State coverage | mhSUN M&E |
| 11 | PHC staff identified who are motivated to provide mental health services | 11.1 Adequate numbers attend training for model needs (4 per PHC), making allowance for attrition | Numbers specified according to model | mhSUN M&E |
| 12 | Psychiatrist able to train 1^0^ and 2^0^ care staff in all mhGAP conditions, & nurses to supervise PHCs | 12.1 Adequate Psychiatrists (5) trained for mhGAP training and supervision in each State [Binary; Yes/No] | Pre and post-test evaluation scores following mhGAP Supervisor training | mhSUN M&E |
| 13 | Adequate on-going management, quality control and supportive clinical supervision in place by psychiatrist in 2^0^ and psychiatric nurses in 1^0^ care | 13.1 Supported supervisory visits happen routinely (1 per month min at each facility)  - Feedback form for the clinic staff about the quality of supervision completed  - Fidelity is adequate | Use of supervision checklist  Qualitative data from PHC staff at the end of the project, about the quality of supervision received  Fidelity measure | mhSUN M&E |
| 14 | Psychiatric nurses, & Medical Officers, able to diagnose, treat & refer people with priority disorders | 14.1 Adherence with mhGAP treatment guideline  14.2 Adequate quality of care reported by clients | ? Facility detection survey  Review of notes by supervisor  Referral forms  Client satisfaction questionnaires and interviews | mhSUN M&E  Research project |
| 15 | CHOs, nurses & CHEWs able to diagnose, treat & refer people with priority disorders | 15.1 Adherence with mhGAP treatment guideline  15.2 Adequate quality of care reported by clients | ? Facility detection survey  Review of notes by supervisor  Referral forms  Client satisfaction questionnaires and interviews | mhSUN M&E  Research project |
| 16 | Existing community structures engaged with programme | 16.1 Identified relevant community resources ready to support clients | Community resource mapping | mhSUN M&E |
| 17 | Community champions able to identify people in need and raise awareness | 17.1 Community champions listed in catchment of each PHC | Community resource mapping | mhSUN M&E |
| 18 | Community is aware of mental illness and availability of services | 18.1 Increase in client use of new services  18.2 Greater knowledge about mental illness and availability of service among key informants  18.3 Awareness activities carried out by community champions / PHC staff | Routine HMIS  Count community awareness indicators such as availability of posters  Focus Groups with community members | Routine HMIS  mhSUN M&E  Research project |
| 19 | People with mental disorders are willing to seek treatment | 19.1 Increase in client use of services | Routine HMIS  Interviews with attending  ? and non-attending clients | Routine HMIS  Research project |
| 20 | People with mental disorders are identified in the community | 20.1 Increase in client use of services to meet coverage targets for each priority disorder | Routine HMIS  Compare with known prevalence rates (-> coverage) | Routine HMIS  Research project |
| 21 | People with mental disorders are diagnosed and treated in PHC including brief psycho-social therapies | 21.1 People with mental disorders are diagnosed and treated in PHC according to mhGAP Guidelines | Routine HMIS  Routine supervision records  Review of notes  Compare with known prevalence rates (-> coverage) | Routine HMIS  mhSUN M&E |
| 22 | 1^0^ staff refer to general hospital and for community services according to mhGAP guidelines. People who are non-adherent are identified | 22.1 PHC staff follow management as outlined in mhGAP  22.2 Non-attendees to follow-up are identified and action is taken according to model | Routine HMIS  Routine supervision records  Review of notes  Referral records  Cohort survey and delineation of pathways to care | Routine HMIS  mhSUN M&E |
| 23 | People with mental disorders are diagnosed and treated in general hospitals, including psychological therapies | 23.1 Hospital staff follow management as outlined in mhGAP  23.2 Personnel have appropriate competence at each level to deliver care according to mhGAP Guidelines | Routine HMIS  Routine supervision records  Review of notes  Referral records  Cohort survey and delineation of pathways to care | Routine HMIS  mhSUN M&E |
| 24 | 2^0^ staff refer to outpatient clinic, 3^0^ care & community services according to mhGAP guidelines. People who are non-adherent are identified | 24.1 Hospital staff follow management as outlined in mhGAP  24.2 Personnel have appropriate competence at each level to deliver care according to mhGAP Guidelines | Routine HMIS  Routine supervision records  Review of notes  Referral records  Cohort survey and delineation of pathways to care | Routine HMIS  mhSUN M&E  Research project |
| 25 | People who are non-adherent are followed up in the community | 25.1 Non-attendees to follow-up are identified and action is taken according to model | Tracked as part of the Cohort | mhSUN M&E |
| 26 | People receive locally available services for social, economic and educational needs | 26.1 Mapped community resources are used appropriately according to client needs | Cohort survey and delineation of pathways to care | mhSUN M&E |
| 27 | 3^0^ services available and people referred back to 1^0^ and 2^0^ care for follow up | 27.1 People access 3^0^ services at FNPH  27.2 FNPH refers appropriate cases for follow-up at general hospital care | Referral records | mhSUN M&E |
| 28 | Services accessible, affordable and acceptable by all | 28.1 Adapted version of mhGAP Guidelines used and trained.  28.2 Model developed with local cultural and service factors in mind  28.3 Clients costs measured | Client satisfaction questionnaire  Costs of care measured, eg using Client Service Receipt Inventory | mhSUN M&E  Research project |
| 29 | People with priority disorders receive treatment as intended for the required duration | 29.1 Number of patients treated (and types of disorders they have) | Cohort survey and delineation of pathways to care | mhSUN M&E  Research project |
| 30 | Improved mental health outcomes for people with mental disorders treated by mhSUN | 30.1 Cohort followed to track outcomes | Cohort survey and delineation of pathways to care | Research project |
| 31 | Increased effective coverage of evidence-based mental health services in mhSUN implementation areas | 31.1 Coverage of services (by priority disorder)  31.2 Quality of care is adequate | Use catchment area populations and prevalence rates (denominator) against service utilization rates (numerator)  Supervision reports  Client satisfaction survey | Routine HMIS  Research project |

*** Responsibility for data collection**

Listed are those primarily responsible

- mhSUN M&E includes local management teams and staff during routine supervision and reporting processes
- HMIS is through mainstream integrated system if possible
- Research project is using independent research assistants and overseen by JE (supervised by OG and MdS)

However, there is much overlap, for example the data from routine HMIS and M&E will be used in research, and research data will be made available for mhSUN reporting.
